# Bowel-Affections of Children

**Published:** 1867-11

**Authors:** N. S. Davis

**Affiliations:** Medical Wards of Mercy Hospital


### BOWEL-AFFECTIONS OF CHILDREN.

*Abstract of a Clinic by Prof.* N. S. Davis, *in Medical Wards of Mercy Hospital. September, 1867.*

Gentlemen:—Here is an infant, about eight months old, laboring under one of the most common and fatal forms of disease that the practitioner is called upon to treat. And though the mother has brought it here unexpectedly, I shall make no apology for occupying your attention with the disease which it illustrates, during the present clinic hour.

You notice the child is pale, emaciated, restless, the eyes a little sunken, the line from the alæ-nasi to the angles of the mouth depressed, making an expression of sadness; and if you examine more closely, you find the skin cool, particularly on the extremities; the pulse small and quick; the muscles flaccid, and the mother will tell you that about two weeks since the child was attacked with serous diarrhœa and vomiting. The latter ceased, or occurred only occasionally, after the first 24 hours, but the diarrhœa has continued to the present time. You will readily recognize the case as one of mild cholera infantum followed by simple diarrhœa; the discharges being thin, varying in color, sometimes green, other times yellow, and again ash-gray, and much of the time very offensive; while the urine is generally scanty. If these symptoms were suffered to continue two or three weeks longer, the child would be reduced to a skeleton; its eyes deeply sunken; its lips pale and thin; the skin in folds or wrinkles on its neck, thighs, and arms; with a feeble pulse, and often husky voice. In this condition, the functions of digestion and assimilation are often entirely suspended, and the patient soon reaches a stage of fatal exhaustion.

The bowel-affections met with in children, every summer, may be arranged into three groups. The first or simplest form of disease commences with mere looseness of the bowels, indicated by from two to six passages daily, more thin and copious than natural, and varying in color from ash-grey to light yellow. It is accompanied by vomiting only occasionally, when the stomach is made too full, and the child often continues cheerful and free from fever, though daily becoming more pale, thinner in flesh, and at times peevish. In this mild form, the disease may continue through the greater part of July and August without the occurrence of structural lesions, and gradually disappear in the early autumn, without any treatment whatever. Many other cases, however, either from a more active grade of irritation in the intestinal mucous membrane, or from a scrofulous constitutional taint, do not thus recover. On the contrary, they are found at the end of from eight to twelve weeks, pale, bloodless, emaciated to a skeleton, the pulse feeble, skin cool, abdomen tumid, appetite often voracious, and bowels moderately loose and, generally, discharges very offensive, with appearances of undigested food. These symptoms indicate such a change in the mucous membrane of the ilium and the mesenteric glands, that the work of assimilation cannot be carried on, and hence most of such cases terminate fatally; though not, in some, until several months have elapsed.

The second class of cases commence more abruptly, with active vomiting and diarrhœa. Everything that is taken into the child's stomach is quickly rejected by vomiting, often accompanied by much retching; the intestinal discharges are frequent, copious, very thin, and generally of a green or yellow color. The eyes rapidly sink in the socket, the extremities become cool, the pulse small and frequent, with paroxysms of great restlessness. In the most severe cases, the little child becomes so rapidly exhausted that complete collapse and death, sometimes, occurs in from 24 to 48 hours. In cases of less severity, after the first 12 or 18 hours, the vomiting either ceases or becomes occasional only, the intestinal discharges less frequent and copious, and a slight febrile reaction takes place; from this time on, the child continues fretful, thirsty for cold drinks, and constantly troubled with indigestion. If it takes milk or other food, much of it either sours in the stomach and is often rejected by vomiting, or it is hurried through the intestines and appears in undigested curds and masses in the stools. The latter become very variable in color, consistence, and composition. In some cases, they continue very thin, green, or yellow, or ash-grey, and containing only slight indications of mucus and epithelium; in others, they are frothy and offensive; and in still others, they are small in quantity, consisting mostly of mucus mixed with undigested food, and variegated in color. Of course the child continues to emaciate, and often reaches a state of fatal exhaustion in from one to six weeks.

The third variety or group of cases met with in practice present dysenteric symptoms from the beginning.

They are feverish, restless, thirsty, with quick pulse, hot skin, irritable stomach, frequent small mucus discharges, sometimes mixed with blood, and accompanied by straining or tenesmus. The disease pursues the same general course as dysentery in the adult.

From these remarks, you will readily perceive that the bowel-affections of children vary much in their symptoms, their pathology, and their results. The essential pathological conditions in the first class of cases are, extreme morbid sensibility of the mucous membrane of the alimentary canal, and relaxation of the same tissue, or diminished tonicity of the capillaries. This causes increased secretion and sometimes effusion from the free surface of the mucous membrane, furnishing the increased discharges, while the function of absorption is, in the same ratio, impaired. Hence, much of the ingesta passes through mixed with the morbid secretions, instead of being appropriated as nourishment.

In the first stage of the second class of cases, the same pathological conditions of the mucous membranes exist, only in a more exaggerated degree. The morbid sensibility and relaxation are such, that the resulting effusion becomes rapid and copious, as indicated by the frequent and copious discharges both up and down. This rapid effusion not only reduces the relative proportion of serous or watery element of the blood, but it also disturbs and detaches more or less of the epithelial covering of the mucous membrane. If the patient does not pass into collapse and death at this stage, congestion takes place in the denuded patches of membrane, and a low grade of inflammatory action follows, with some general febrile reaction, constituting the second stage of the attack. If not controlled, the inflammation thus begun will persist, in many cases, until ulceration and emaciation reach an extent fatal to the little sufferer.

Pathologically, the third group of cases do not differ from cases of ilio-colitis and recto-colitis, in adults. In regard to the causes of these different grades of bowel-affections, a few words are necessary.

Gentlemen, in nine cases out of ten you will be told by the mother or nurse, that the child is *teething;* and, I am sorry to add, that the same thing will be alleged by a large portion of the profession, as though the simple growth of the child's teeth, and especially their exit through the gums, was a sufficient cause of all these cases. So fixed is this notion in the minds of the people, that thousands of infants are sacrificed every year needlessly, especially among the poorer classes of society. The mother will present you her child for the first time, already emaciated to a skeleton; its eyes deeply sunken; lips thin, pale, and retracted; the skin hanging in folds around its arms and legs; the pulse feeble; the bowels moving from ten to twenty times a-day; with entire suspension of digestion and assimilation.

You ask how long the child has been sick; the mother will answer generally, "three or four weeks, but not to say right sick until the last few days." You ask her what she has been doing for the child all that long time, and she will say, "nothing, except two or three times a dose of castor-oil." You ask why she neglected the child so long, until it is, perhaps, past recovery, and she will reply promptly, "oh! it was *teething*, and she did not think the disturbance of the stomach would signify anything."

Now, it is just this idea of the effects of teething that cause not less than 300 infants to die from neglecting these bowel-affections, in Chicago alone, every summer. And, yet, nothing is more certain, than that the growth of the teeth exerts no influence whatever, as a cause of these affections in children; as the following facts will show:

1st. The attacks occur at all periods of age, from birth to two or two and a-half years: that is, before the growth of the teeth has made the slightest impression upon the gums, and after all are through that will be for some years.

2d. When an attack has commenced, neither the spontaneous exit of one or more teeth through the gum, nor the incision of the gum, affords any perceptible relief to the intestinal symptoms.

I must caution you against one source of error here. It often happens, that one of these little children is presented to the physician, who carefully examines and scarifies the gums, and, at the same time, prescribes some appropriate medicine to allay the intestinal irritation. The child takes the medicine, becomes better, and, of course, the cutting of the gums is credited with the greater part of the resulting benefit, while, in reality, it exerted no influence whatever.

3d. I have carefully examined the mouths of several hundred infants in the first stage of bowel-affections, and have not found either redness, heat, tenderness, or any other sign of irritation in the gums, in five per cent of the whole number.

But, gentlemen, you will be told, with great emphasis, that "it must be the teeth, for the child is constantly putting its fingers in its mouth, and is biting on everything it can get hold of." Those who make this assertion so gravely forget that the child had been doing precisely the same thing ever since the hour of its birth; and that the act was one of the best evidences that the mouth and gums were free from soreness or morbid sensibility. No child will be found cramming its fingers into a sore mouth, or willingly biting upon a tender or sensitive gum.

4th. The well-known fact, that the diseases in question are restricted in their prevalence to certain geographical limits, and to certain seasons of the year, totally disprove the agency of teething as an efficient cause. Of course, children "cut their teeth" as much in one country as another—as much in January as in July. In this city, for instance, there were the same ratio of children "cutting their teeth" in January last, as in the July following. Yet, in the first month named, there were scarcely more than ten deaths from bowel-complaints in young children, while in the last named month there were near three hundred.

This brings us to the real causes of these affections, namely: high temperature, with impure air, in such localities as give a wide range of temperature between the extremes of winter and summer. In all the populous cities, and in most of the malarious country districts, in what is called the temperate zone of this continent, the first six or seven consecutive days of hot, sultry weather, occurring at the commencement of each summer, always starts the prevalence of diarrhœa and cholera-morbus among the young children. The coincidence between the high atmospheric temperature and the prevalence of bowel-complaints is so constant, from the beginning to the end of the summer, that no careful observer will doubt their relation, as *cause* and *effect*. To develope a severe prevalence of this class of diseases, the high temperature must act conjointly with such atmospheric impurities as are found in all large cities and in some malarious districts.

*Treatment.*—The principles that have guided us in the treatment of the various forms of intestinal disease in children, are easily inferred from the explanations already given, concerning their pathology. In the first stage of the two first varieties described, the indications are, to allay the morbid excitability of the mucous membrane, and to restore the proper tone or contractility to its capillary and secretory structures. At a later stage, when, from the continued drain of excessive intestinal discharges the excretory functions, especially those of the skin and kidneys, are greatly impaired or even wholly suppressed, a third indication is, to add such remedies as will aid in restoring both assimilation and secretion. In the simplest form of intestinal disease, as represented in the first class or group of cases described, the union of a simple anodyne and tonic; the one to allay the morbid excitability, and the other to improve the tonicity; fulfils the whole therapeutic indication in the early stage. A formula that I have used with much satisfaction for many years, consists of:

℞. Aromat. Sulph. Acid, ------------------ ʒij.

Sulph. Magnesia, ------------------------ ʒij.

Tinct. Opii, ------------------------------ ʒij.

Simple Syrup, -------------------------

Mint Water,---------------------------āā ℥j.

Mix.

Of this, I give, to a child six months old, 10 or 12 drops in a teaspoonful of sweetened water, every three, four, six, or eight hours, according to the frequency of the discharges, and continue their use until the discharges are reduced to one a-day, and natural in appearance. If it should happen that the discharges cease altogether for twenty-four or even thirty-six hours, do not immediately administer a cathartic, and thereby undo all you have accomplished, but simply suspend the use of the restraining medicine, and wait until the bowels are moved spontaneously. If, when this takes place, the discharge is more natural and without a tendency to frequent repetition, the further use of the drops may be restricted to one dose at night, or night and morning.

It was this prescription that I directed for the little child before you, when it was first brought to me, three days since. Its discharges are already much less frequent and more healthy, and we shall continue the remedy with the confident expectation that its recovery will be complete in three or four days more. Another formula, which I frequently use for the same purpose, is as follows:

| | | |
|---|---|---|
| ℞. | Sub. Nit. Bismuth,------------------- | 30 grs. |
| | Pulv. Opii, --------------------------- | 2 grs. |
| | White Sugar,-------------------------- | 40 grs. |

Mix. Divide into 15 powders, of which one may be given to a child one year old, every three, four, or six hours, until the discharges are reduced to their normal frequency.

Whether this or the preceding formula is used, you should inquire carefully concerning the urinary secretion, and if it is in any degree scanty, a few drops of nitrous ether should be given between each of the doses of other medicine. When these cases of simple summer diarrhœa have already become chronic, with much emaciation and debility, I have often obtained the most favorable results from the use of the following:

| | | |
|---|---|---|
| ℞. | Erigeron Canadensis, ----------------- | ℥ss. |
| | Tannate of Quinine,------------------ | 20 grs. |
| | Sulph. Morph.,------------------------ | 1 gr. |

Mix. Pour on it half a pint of boiling water, stir it up well while hot. When cold, give of this infusion, to a child one year old, a teaspoonful every three, four, or six hours, according to the frequency of the discharges.

In the more severe class of cases, such as described in the second group, constituting cholera infantum proper, if called while the vomiting and purging are still active, I generally make two prescriptions, as follows:

℞. Bicarb. Soda,.............................. ʒj.

Sulph. Morph.,.............................. 1 gr.

Aqua Menth.,.............................. ℥ij.

Mix, and give, to a child one year old, 10 drops in a teaspoonful of sweetened water, *immediately* after every turn of vomiting; and,

℞. Hydrarg. Chlorid. Mite,.............. 4 grs.
Pulv. Opii,.............................. 1 gr.
Sacchar. Alba,.............................. 20 grs.

Mix. Divide into 8 powders, and give one every three or four hours, until the discharges cease.

After the active vomiting and purging are once checked, if the discharges from the bowels continue too frequent and thin, the subsequent treatment may be the same as already detailed for simple diarrhœa. But if the active stage gives place to a degree of febrile reaction accompanied by small mucous discharges, whether mixed with blood or not, then the following formula becomes far more efficient than any other that we have used:

℞. Ol. Terebinth.,.............................. ʒij.

Tinct. Opii,.............................. ʒij.

Pulv. G. Arabic,..............................

White Sugar,.............................. āā ʒiij.

Rub together and add,

Mint Water,.............................. ℥ij.

Mix, and give, to a child 12 or 15 months old, 15 drops every three or four hours, until the discharges are checked, and then continue at longer intervals, until they become natural.

The third class of cases were represented to be dysenteric from the beginning, and, hence, they must be treated as such. But the clinic hour has already expired, and you must be dismissed for the present.

